# The Efficacy of the SinHumo App Combined With a Psychological Treatment to Quit Smoking: A Randomized Clinical Trial

**DOI:** 10.1093/ntr/ntae053

**Published:** 2024-03-27

**Authors:** Ana López-Durán, Carmela Martínez-Vispo, Daniel Suárez-Castro, María Barroso-Hurtado, Elisardo Becoña

**Affiliations:** Smoking and Addictive Disorders Unit, Faculty of Psychology, Department of Clinical Psychology and Psychobiology, University of Santiago de Compostela, Santiago de Compostela, Spain; Smoking and Addictive Disorders Unit, Faculty of Psychology, Department of Clinical Psychology and Psychobiology, University of Santiago de Compostela, Santiago de Compostela, Spain; Smoking and Addictive Disorders Unit, Faculty of Psychology, Department of Clinical Psychology and Psychobiology, University of Santiago de Compostela, Santiago de Compostela, Spain; Smoking and Addictive Disorders Unit, Faculty of Psychology, Department of Clinical Psychology and Psychobiology, University of Santiago de Compostela, Santiago de Compostela, Spain; Smoking and Addictive Disorders Unit, Faculty of Psychology, Department of Clinical Psychology and Psychobiology, University of Santiago de Compostela, Santiago de Compostela, Spain

## Abstract

**Introduction:**

This study assessed the efficacy of the SinHumo App combined with a cognitive-behavioral smoking cessation treatment on 12-month follow-up abstinence, compared with the same smoking cessation treatment and a control App.

**Aims and Methods:**

A sample of 288 treatment-seeking people who smoke were randomized: SinHumo App plus smoking cessation treatment (*n* = 140) and control App plus smoking cessation treatment (*n* = 148). The primary outcome was 7-day point prevalence abstinence (PPA) at the 12-month follow-up. Secondary outcomes were abstinence rates at the end of the intervention and 3- and 6-month follow-ups, cigarette per day (CPD) reduction over the 12-month follow-up, intervention engagement, and satisfaction.

**Results:**

Intention-to-treat analyses showed nonsignificant differences in self-reported 7-day PPA at the 12-month follow-up (37.1 and 42.6%, respectively; OR = 0.80). No significant differences were found in abstinence at the end of the treatment (68.6 vs. 62.8%) nor on 7-day PPA at 3- (35.7 vs. 45.9%) and 6-month (35.0 vs. 41.2%) follow-up. Complete case and multiple imputation analyses yielded similar results for abstinence outcomes. A significant reduction in CPD across the 12-month follow-up in the subsample of participants who smoked was observed, but nonsignificant differences between conditions were found. Higher engagement with the SinHumo App was a significant predictor of 12-month abstinence. Satisfaction with the intervention was high and similar in both groups.

**Conclusions:**

High abstinence rates over the 12-month follow-up and satisfaction were found in both conditions. The inclusion of the SinHumo App did not improve abstinence rates in the intervention.

**Implications:**

Scarce research has examined the long-term efficacy of smoking cessation treatments, including Apps, to support the quitting process. The present randomized controlled trial contributes to the existing literature about including information and communication technologies in behavior change interventions. The development of effective smoking cessation apps and information and communication technologies-based interventions is crucial for reducing the prevalence of smoking, as these interventions have the potential to reach a large number of people who smoke and reduce access-related barriers to treatment.

## Introduction

Tobacco use continues to be the leading preventable cause of mortality and morbidity worldwide.^1^ Over the past 50 years, effective psychological treatments have been developed to quit smoking.^2,3^ The *U.S. Preventive Services Task Force Recommendation Statement*^4^ points out that behavioral interventions contribute significantly to smoking cessation, indicating that psychological treatment is the first choice. Despite the positive results of psychological treatments to quit smoking, relapse into tobacco use, as in other addictions, is common.^5^ Blyth et al.^6^ suggest that using digital-based support in smoking cessation treatments could be a venue to reduce relapse rates. These digital tools could provide tailored and personalized support to cater to the specific needs and contexts of people trying to quit, making smoking cessation treatments more attractive and engaging. In recent years, interventions have been developed based on Apps to quit smoking,^7,8^ mainly in self-help formats.^9^ However, many of these App interventions do not follow evidence-based guidelines to quit smoking, and only a few have been assessed.^10^ This has led to systematic reviews and meta-analyses analyzing the effectiveness of smoking cessation Apps, usually including a reduced number of studies. For instance, the Cochrane systematic review conducted by Whittaker et al.^7^ only included five randomized controlled trials exploring smoking cessation Apps, which limits the certainty of the outcomes. Recently, Guo et al.^11^ conducted a meta-analysis including nine randomized controlled trials and concluded that stand-alone Apps for smoking cessation did not improve abstinence outcomes. However, the use of Apps could be considered as a complement to traditional smoking cessation treatments and for relapse prevention.^12^

The combination of psychological treatment to quit smoking and Apps is a novel line of research towards which we must direct our efforts, given the strong impact that rapid technological progress is having on our society.^13^ Different authors point out that incorporating an App as a complement to a smoking cessation treatment could increase the intensity of the treatment and improve abstinence rates.^14^ Adding the use of complementary Apps to traditional smoking cessation treatments has numerous advantages, including: Ease of use anywhere, anytime; the delivery of information and messages regardless of the patient’s location; the ability to tailor messages to the user’s characteristics; the ability to send messages in real-time and the facilitation of social support through the user’s contact with other people.^15,16^ These Apps allow obtaining real-time data on treatment compliance and the performance of different tasks (eg, self-reports of tobacco use); they improve efficiency, as practitioners can check their progress through a website before the sessions; they increase user motivation, are more attractive, and are preferable to paper forms (Dahne et al., 2018). Despite the opportunities offered by using Apps to support face-to-face treatments for smoking cessation, scarce research has been conducted to date.^17,18^

The main objective of the present study was to evaluate the efficacy of the SinHumo smartphone App (iOS and Android) combined with face-to-face cognitive-behavioral smoking cessation treatment, compared with a control group that receives the same smoking cessation treatment and a control App. Specifically, the primary outcome was the 7-day point prevalence abstinence (PPA) at the 12-month follow-up. Secondary outcomes were: (1) abstinence at the end of the intervention and at the 3- and 6-month follow-ups, (2) reduction of ≥ 50% of cigarettes smoked per day (CPD) from baseline to the 12-month follow-up, (3) prolonged abstinence with lapses, (4) intervention engagement, and (5) satisfaction with the intervention.

## Materials and Methods

### Design and Setting

This was a double-arm, single-blind, randomized controlled design study to assess the efficacy of a cognitive-behavioral intervention for smoking cessation combined with the SinHumo App at the end of treatment and at the 3-, 6-, and 12-month follow-ups. The study was approved by the Institutional Ethics Review Board of the University of Santiago de Compostela (USC-15/2020) and registered at clinicaltrials.gov (NCT04765813).

We used the statistical program G* power to determine the sample size.^19^ Estimating a 20% difference between conditions at 1 year of follow-up and a statistical power of 90% with a significance level of 0.05, the minimum sample needed would be 264 participants (132 per treatment condition).

### Participants

**Figure 1.**
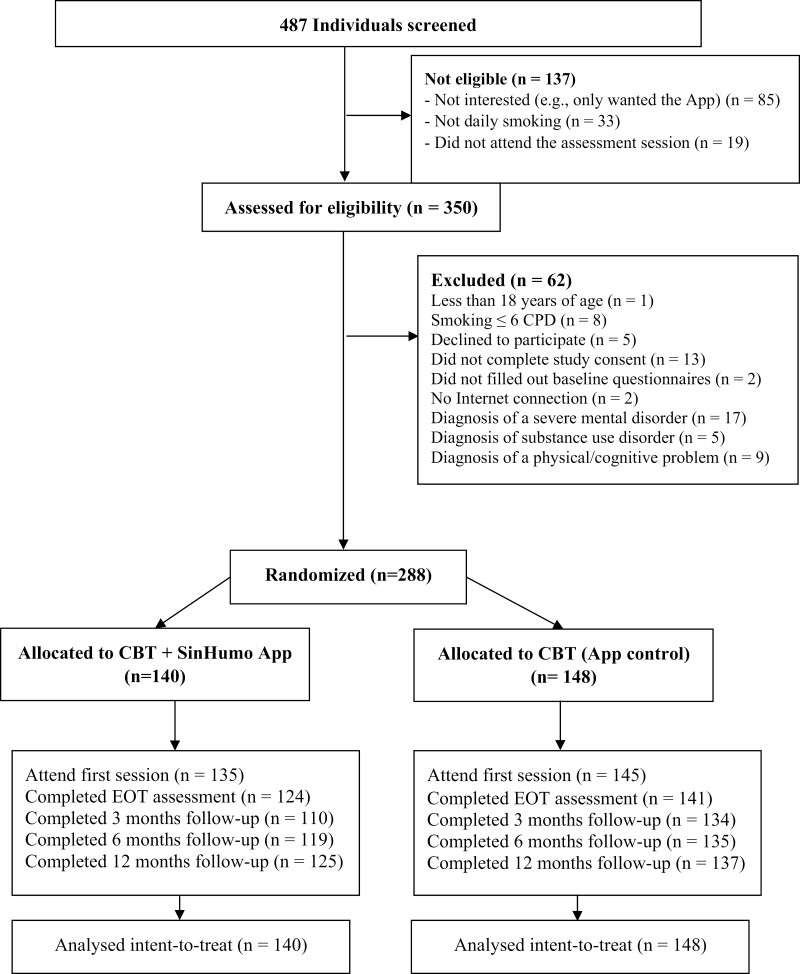
Participant flow diagram. CBT = cognitive-behavioral treatment; CPD = cigarettes per day.

The sample of this randomized controlled trials study comprised 288 treatment-seeking people who smoke. They asked for cessation treatment at the Smoking and Addictive Disorders Unit of the Faculty of Psychology of the University of Santiago de Compostela from September 2020 to October 2022 (Figure 1). Participants were recruited through posters in healthcare centers and hospitals, referred by services of the healthcare system or other professionals (eg, dentists), publications on the Smoking Cessation Unit’s social networks, or word of mouth. None of the participants received financial compensation for their participation. Before taking part in the study, the informed consent of participants was obtained.

The inclusion criteria to be part of the study were to be 18 years of age or older, to want to participate in treatment to quit smoking, to have a minimum consumption of six cigarettes per day before the start of treatment, to duly complete all pretreatment assessment questionnaires, to have a valid e-mail address and a smartphone (Android or iOS) and to be willing to use it throughout the treatment, and to obtain the informed consent. The exclusion criteria were: Having a diagnosis of a severe mental disorder (bipolar disorder and/or psychotic disorder); concurrent addictive disorder of other substances (cannabis, cocaine, and heroin); smoking exclusively rolling tobacco, cigars, or cigarillos (due to the impossibility to follow the nicotine fading procedure); having completed an effective psychological or pharmacological treatment (nicotine gum or patches, bupropion, and varenicline) to quit smoking during the previous year; suffering from a pathology that implies a high risk to the life of the person, which would require immediate intervention (eg, recent myocardial infarction, pneumothorax); having visual difficulties that prevent the proper use of the App; and not attending the first group treatment session.

The experimental group received a cognitive-behavioral treatment (CBT) to quit smoking with the SinHumo App, whereas the control group received a CBT to quit smoking with a control App. Participants were randomized in blocks of four to six participants (1.1. ratio) to the experimental (CBT + SinHumo App) or control group (CBT + Control App) according to a computer-generated randomization list (Excel—Microsoft). All participants within a group were allocated to the same treatment condition (experimental or control) to prevent possible contamination.

### Procedure

Treatment sessions and follow-ups were conducted by general health psychologists trained to deliver the CBT described above. Research staff supervised professionals’ adherence to the study protocol procedures of the assessment and intervention.

Participants meeting the inclusion criteria were individually assessed through a face-to-face interview, and filled in a set of questionnaires (described below). After this initial assessment, participants were randomly assigned to study conditions. The eight treatment sessions were then applied in both conditions in group format (group size: Four–six). Participants of each group started and finished the eight sessions together. During the first treatment session, each participant downloaded the App (SinHumo App vs. control App) from iTunes or Google Play guided by the professional, and received an individual access code to activate the App. Access to the App was provided from the start of the face-to-face treatment until the 12-month follow-up. Participants were unaware of their allocated group (SinHumo App vs. control App).

An end-of-treatment assessment was conducted in the last treatment session (session 8), and subsequently, follow-up assessments were conducted at 3-, 6-, and 12 months in both treatment conditions. Due to the situation generated by the coronavirus pandemic in 2020 (COVID-19) and the social distancing measures that were established, it was not possible to biochemically validate abstinence. Therefore, abstinence was only self-reported. Smoking cessation literature suggests that when in-person contact is not feasible, self-reported abstinence seems to be a reliable measure.^20,21^

### Intervention Conditions

The protocol used, the characteristics of the intervention, and the detailed description of the SinHumo App have been previously published.^22^ The treatment carried out in both conditions was a psychological CBT to quit smoking.^23,24^ It consists of eight sessions in group format, at the rate of 1 weekly session lasting 1 hour, for 8 weeks. The quit day is established in the fifth session, but if participants want to make a quit attempt before, they are encouraged to do it. The components are as follows: (1) therapeutic contract, (2) smoking behavior self-recording and graphic representation of consumption, (3) tobacco information, (4) nicotine fading technique, (5) stimulus control, (6) activities to avoid withdrawal symptoms, (7) behavioral activation components, and (8) relapse prevention strategies (problem-solving training, anxiety and anger management, physical exercise, weight management, etc.).

In the experimental treatment condition (CBT + SinHumo App), the previous intervention was complemented with the SinHumo App with therapeutic components that were used during treatment (self-recording of cigarettes, access to session materials, performance of behavioral activation activities, intersessional notifications reinforcing the achievement of goals, and motivational notifications) and during follow-up. The SinHumo App content was aligned with the objectives and contents of each session guided by the professionals. The components during follow-up were adapted to the participant’s smoking status: (1) abstinence, (2) relapse (quitting smoking at the end of treatment, but relapsed during follow-up), or (3) smoking (not quitting at the end of treatment), both at the end of treatment and at the follow-ups. Some of these components are: Behavioral and cognitive tips for maintaining abstinence or smoking cessation, strategies for coping with the urge to consume, motivational strategies, gains and achievements attained since quitting smoking (in the case of people who are abstinent), or different notifications depending on their status, among others.

In the control treatment condition (CBT + control App), the same treatment as the experimental condition was used, but in this condition, the Control App only had the self-recording of cigarettes component and the session materials in PDF format. This App was available during treatment and follow-up periods.

Follow-ups were conducted 3, 6, and 12 months after treatment ended. Both treatment sessions and follow-ups were conducted via video call using the “Microsoft Teams” platform.

### Measures

#### Smoking Questionnaire

This comprises 59 items that collect baseline information on sociodemographic variables and aspects related to smoking behavior.^25^

#### Fagerström Test for Cigarette Dependence

This test presents six items with two to four response alternatives and a cutoff point of 6 for dependence. This questionnaire was administered in the initial assessment session. ^26–28^

#### Daily Self-records of Cigarette Consumption

This records the number of cigarettes, the time at which the participant smokes each cigarette, the pleasure it gives them (from 0 to 10, with 0 being the minimum pleasure and 10 being the maximum), and their situation when they smoke.

#### End-of-Treatment Evaluation Questionnaire

This is a self-report that collects information about the date of abandonment, confidence in remaining abstinent, perceived social support, and physical and psychological improvement/worsening since the start of treatment. This questionnaire was administered at the end of the eight treatment sessions.

#### Customer Satisfaction Questionnaire-8

This is an eight-item self-report instrument that assesses overall satisfaction with the treatment services administered after the end of the intervention.^29,30^

#### Follow-up Questionnaire

This is a self-report that collects data on abstinence and/or relapse at the 3-, 6-, and 12-month follow-ups. This questionnaire has two versions: One for participants who are abstinent (ie, whether they have smoked any puff or cigarette in the last 24 hours, 7 days, 30 days, and 6 months or since the end of treatment, how long they have been abstinent and the date they smoked their last cigarette) and other one for participants who are smoking (ie, number of cigarettes they smoke per day and tobacco brand, whether they have made other quit attempts).^31^

### Outcomes

Smoking outcomes were defined as recommended by the Society for Research on Nicotine and Tobacco Treatment Research Network.^32^

The primary outcome was 7 days PPA (“not even a puff”) at the 12-month follow-up. The secondary outcomes were:

- Seven days PPA (“not even a puff”) at the 3- and 6-month follow-ups; 30 days PPA (“not even a puff”) at the 6- and 12-month follow-ups; and 24 hours abstinence at the end of treatment.
- Prolonged abstinence with lapses (no more than 5 cigarettes during the 6- or 12-month follow-ups).
- Reduction of ≥50% of cigarettes smoked per day (CPD) from baseline to the 12-month follow-up.
- Engagement with the intervention, defined as the number of sessions attended by participants, and engagement with the App, defined as the number of days of use through the treatment and 12-month follow-up period.
- Satisfaction with the intervention.

### Statistical Analysis

Descriptive analyses were conducted to characterize the total sample. Chi-square tests and Student *t-tests assessed differences between the study conditions (CBT* + SinHumo App vs CBT + control App) on demographics and smoking-related variables.

The primary and secondary abstinence-related outcomes were analyzed using two approaches: (1) intention-to-treat analyses (including all randomized participants and considering missing data as smoking) and (2) complete-case analyses (including only those participants reporting their smoking status). As a sensitivity analysis, multiple imputation analyses were also conducted to handle missing data. We included in the imputation model the following variables: treatment condition, demographics (sex, age, and educational level), cigarette dependence (Fagerström Test for Cigarette Dependence), and history of depression treatment. Twenty imputed datasets were generated, and pooled results were reported. Binary logistic regression analyses were conducted to examine abstinence at each time-point assessment (at the end of treatment, at the 3-, 6-, and 12-month follow-ups) unadjusted and adjusted by the following covariates: Sex, age, Fagerström Test for Cigarette Dependence, and history of depression treatment. Although these variables did not differ between conditions at baseline, adjusted analyses were conducted in order to account for their potential effect on cessation success based on previous literature.^33–35^ Secondary-related analyses were conducted to assess treatment satisfaction differences according to treatment conditions.

SPSS version 29 was used for statistical analysis. The value of the significance level was set at 0.05.

## Results

The total sample of participants included 288 people who smoked daily (62.5% women; 180/288; Mean age = 45.80, SD = 10.63 years). Table 1 shows demographics and smoking-related variables at baseline for the total sample and each treatment condition. Over half of the sample had university studies (52.1%; 150/288). Regarding smoking, participants smoked an average of 18.63 cigarettes per day (SD = 8.95), the mean of years smoking was 26.66 (SD = 10.91) ranging from 1 to 59 years, and 38.2% (110/288) obtained a score of six or higher in the Fagerström Test for Cigarette Dependence. No significant group differences were found in these variables.

**Table 1.** Participants’ Sociodemographics and Smoking-Related Variables at Baseline

	Total sample (*N* = 288)	CBT + SinHumo App (*n* = 140)	CBT + control App (*n* = 148)			
	Mean/*n* (SD/%)	Mean/*n* (SD/%)	Mean/*n* (SD/%)	χ^2^	*t*	*p*
Age (years)	45.80 (10.63)	45.95 (11.57)	45.66 (9.69)		0.235	.815
Sex				0.015		.903
Female	180 (62.5)	88 (62.9)	92 (62.2)			
Marital status				0.183		.669
Married/living with a partner	130 (45.1)	65 (46.4)	65 (43.9)			
Single/divorced/widowed	158 (54.9)	75 (53.6)	83 (56.1)			
Education				2.364		.307
< HS diploma	41 (14.2)	21 (15.0)	20 (13.5)			
HS diploma or GED	97 (33.7)	41 (29.3)	56 (37.8)			
University	150 (52.1)	78 (55.7)	72 (48.6)			
Current work situation				0.886		.347
Working (yes)	196 (68.1)	99 (70.7)	97 (65.5)			
CPD	18.63 (8.95)	18.71 (9.34)	18.55 (8.60)		0.152	.880
Age began daily smoking	18.22 (5.12)	17.87 (4.74)	18.55 (5.45)		−1.120	.264
Years smoking	26.66 (10.91)	27.11 (11.42)	26.23 (10.43)		0.681	.496
FTCD	4.82 (2.10)	4.69 (2.19)	4.93 (2.01)		−0.968	.334
Cigarette dependence (FTCD ≥ 6)	110 (38.2)	54 (38.6)	56 (37.8)	0.016		.898

Abbreviations: CBT = cognitive-behavioral treatment; CBT + SinHumo App = CBT plus smartphone App; CPD = Cigarettes smoked per day; HS = high school; GED = general education diploma; FTCD = Fagerström Test for Cigarette Dependence.

Regarding retention rates, 92.0% (265/288) of the participants provided data at the end of the intervention (CBT + SinHumo App, 88.6% [124/140] vs. CBT + control App, 95.3% [141/148], *p = *.036), 84.7% (244/288) at the 3-month (CBT + SinHumo App, 78.6% [110/140] vs. CBT + control App, 90.5% [134/148], *p* = .008), 88.2% (254/288) at the 6-month (CBT + SinHumo App, 85.0% [119/140] vs. CBT + control App, 91.2% [135/148], *p* = .102), and 91.0% (262/288) at the 12-month follow-up (CBT + SinHumo App, 89.3% [125/140] vs. CBT + control App, 92.6% [137/148], *p* = .331; Figure 1).

### Smoking-Related Outcomes

For the primary outcome of 7-day PPA at the 12-month follow-up, no significant group differences were found (CBT + SinHumo App: 37.1 % [52 out of 140] vs. CBT + control App: 42.6% [63 of the 148]; odds ratio [OR], 0.80; 95% CI [0.50, 1.28], *p* = .348). Results were similar when examining complete case data with abstinence rates of 41.6% [52 out of 140] in the CBT + SinHumo App condition and 46.0% [63 out of 148] in the CBT + control App condition (OR = 0.84; 95% CI [0.51, 1.37], *p* = .475).

Regarding abstinence-related secondary outcomes, no significant group differences were observed at the end of the intervention or the 3- and 6-month follow-ups (Table 2) when following the intention-to-treat (ITT) approach. However, in the complete case analyses, the group of CBT + SinHumo App obtained significantly higher abstinence rates than the CBT + control App group at the end of treatment (77.4% [96 out of 140] vs. CBT + control App: 66.0% [93 out of 148]; OR = 1.77; 95% CI [1.03, 3.06], *p* = .040). No significant group differences were found at the 3- and 6-month follow-ups for the complete case analyses. When regression analyses were adjusted by covariates, similar data were obtained (Table 2). Multiple imputation data analyses also showed nonsignificant group differences (Supplementary Material).

**Table 2.** Self-Reported Abstinence Rates by Treatment Condition

	Treatment Group		Odds Ratio (95% CI)	*p*	AOdds Ratio (95% CI)	*p*
	CBT + SinHumo App (*n = *140)	CBT + control App (*n* = 148)				
Outcome variable (ITT)	% (*n*)	% (*n*)				
7-day PPA at 12-month follow-up	37.1 (52)	42.6 (63)	0.80 (0.50; 1.28)	.348	0.76 (0.47; 1.24)	.271
30-day PPA at 12-month follow-up	32.9 (46)	40.5 (60)	0.72 (0.44; 1.16)	.177	0.68 (0.41; 1.11)	.124
7-day PPA at 6-month follow-up	35.0 (49)	41.2 (61)	0.77 (0.47; 1.24)	.278	0.73 (0.45; 1.19)	.202
30-day PPA at 6-month follow-up	34.3 (48)	38.5 (57)	0.83 (0.52; 1.35)	.456	0.79 (0.49; 1.30)	.359
7-day PPA at 3-month follow-up	35.7 (50)	45.9 (68)	0.65 (0.41; 1.05)	.078	0.62 (0.38; 1.01)	.055
End of treatment abstinence	68.6 (96)	62.8 (93)	1.29 (0.79; 2.10)	.306	1.21 (0.73; 2.00)	.472
Outcome variable (complete case)	% (*n*)	% (*n*)				
7-day PPA at 12-month follow-up	41.6 (52)	46.0 (63)	0.84 (0.51; 1.37)	.475	0.81 (0.49; 1.33)	.398
30-day PPA at 12-month follow-up	36.8 (46)	43.8 (60)	0.75 (0.46; 1.23)	.249	0.71 (0.43; 1.18)	.186
7-day PPA at 6-month follow-up	41.2 (49)	45.2 (61)	0.85 (0.52; 1.40)	.520	0.82 (0.49; 1.37)	.449
30-day PPA at 6-month follow-up	40.3 (48)	42.2 (57)	0.93 (0.56; 1.53)	.761	0.90 (0.54; 1.51)	.695
7-day PPA at 3-month follow-up	45.5 (50)	50.7 (68)	0.81 (0.49; 1.34)	.809	0.79 (0.47; 1.33)	.376
End of treatment abstinence	77.4 (96)	66.0 (93)	1.77 (1.03; 3.06)	.040	1.72 (0.97; 3.06)	.063

CBT = Cognitive-Behavioral Treatment; CBT + SinHumo App = CBT plus smartphone App; ITT = Intention to Treat; PPA = Point Prevalence Abstinence; End of treatment abstinence = achieving at least 24 hours of abstinence in the last treatment session (session 8); OR = Odds Ratio; AOR = Adjusted Odds Ratio; Smoking status coded as smoking = 0, abstinence = 1.

We also assessed prolonged abstinence with lapses (no more than five cigarettes during the 6- or 12-month follow-ups). When using an ITT approach, prolonged abstinence at the 6-month follow-up was 29.3% for the CBT + SinHumo App condition versus 35.1% for the CBT + control App, and at 12 months, it was 24.3% for the CBT + SinHumo App condition versus 31.1% for the CBT + control App. Nonsignificant group differences were found. Upon examining complete cases, a similar pattern was found: At the 6-month follow-up, prolonged abstinence was 34.5% for the CBT + SinHumo App condition versus 38.5% for the CBT + control App, and at 12 months, it was 27.2% for the CBT + SinHumo App condition versus 33.6% for the CBT + control App.

Cigarette smoking reduction (≥50%) from baseline to the 12-month follow-up in the subsample of non-abstinent participants (*n* = 173) was also examined. Nonsignificant group differences were found when considering the ITT approach (CBT + SinHumo App: 25.0 % [22 out of 88] vs. CBT + control App: 16.5% [14 out of 85]; OR = 1.89; 95% CI [0.85, 4.21], *p* = .118) or the complete case data (CBT + SinHumo App: 30.1 % [22 out of 73] vs. CBT + control App: 18.9% [14 out of 74]; OR = 1.66; 95% CI [0.77, 3.58], *p* = .197). Finally, to test changes in the mean number of CPD within subjects in each condition, repeated ANOVA was conducted using the Greenhouse–Geisser F (F_GG_) correction in the subsample of people who smoke at the 12-month follow-up. Data showed a significant reduction in CPD over 1 year (*F*_*GG*_ = 54.825, *p* < .001, η_*p*_^*2*^ = 0.243), but no significant group differences were found, *F*(1, 171) = 0.983, *p = *.323.

### Engagement-Related Outcomes

Engagement with the intervention, defined as the number of sessions attended by participants, was similar between conditions (CBT + SinHumo App, *M* = 6.11; SD = 2.30; vs. CBT + control App, M = 5.92; SD = 2.24; *t *= 0.73; *p = *.466). The number of sessions attended by participants was a significant predictor of abstinence outcomes at 12-month follow-up in both study conditions (CBT + SinHumo App, OR = 1.65; 95% CI [1.29, 2.10], *p < *.001; CBT + control App, OR = 1.26; 95% CI [1.06, 1.48], *p* = .007).

Regarding App use, which was defined as the number of days of use through the treatment and 12 months follow-up period, participants with the SinHumo App had greater mean days of use than those with the control App (CBT + SinHumo App, *M *= 37.90; SD = 28.43; vs. CBT + control App, *M *= 26.82; SD = 20.89; *t *= 3.68; *p < *.001). Finally, the number of days of app use was a significant predictor of 12-month cessation success in the CBT + SinHumo App condition (OR = 1.02; 95% CI [1.01, 1.03], *p = *.003), whereas in the control App condition was not significant (OR = 1.00; 95% CI [0.99, 1.02], *p* = .746).

### Satisfaction Outcomes

Overall, participant’s satisfaction with the intervention assessed with the customer satisfaction questionnaire-8 was high for the total sample (*M* = 30.58, SD = 2.57; maximum score of the scale 32), and no significant group differences were found (CBT + SinHumo App; *M* = 30.70, SD = 2.28; CBT + control App: *M* = 30.46, SD = 2.81, *t* = 0.735, *p* = .463). More specifically, to the question, “Overall, how satisfied are you with the service you received?” 86.2% of the participants in the CBT + SinHumo App condition and 84.7% in the CBT + control App group reported being very satisfied, and no significant group differences were found (χ^2^ = 0.923, *p* = .630) (Table 3).

**Table 3.** Client Satisfaction Questionnaire (CSQ-8) Rating by Treatment Condition

	CBT + SinHumo App (*n = *116)	CBT + control App (*n = *131)	*t*	*p*
	*M* (SD)	*M* (SD)		
How would you rate the quality of service you received?	3.90 (0.31)	3.86 (0.37)	0.78	.434
Did you get the kind of service you wanted?	3.84 (0.37)	3.81 (0.43)	0.53	.597
To what extent has our service met your needs?	3.78 (0.42)	3.71 (0.47)	1.16	.249
If a friend needed similar help, would you recommend our service to them?	3.91 (0.29)	3.90 (0.35)	0.11	.915
How satisfied are you with the amount of help you received?	3.85 (0.36)	3.84 (0.41)	0.28	.779
Did the services you received help you to deal more effectively with your problems?	3.72 (0.49)	3.68 (0.54)	0.55	.586
Overall, how satisfied are you with the service you received?	3.86 (0.35)	3.84 (0.39)	0.48	.635
If you were to seek help again, would you come back to our service?	3.86 (0.41)	3.82 (0.49)	0.65	.517

CSQ-8 items range from one to four, with higher scores indicating greater satisfaction with the treatment received.

## Discussion

This study was a randomized controlled trial examining the efficacy of a smartphone App combined with a face-to-face manualized cognitive-behavioral smoking cessation treatment compared with the same CBT intervention with a control App. Abstinence was analyzed over 1 year (at the end of treatment and at the 3-, 6-, and 12-month follow-ups). ITT analyses showed no significant differences in abstinence rates at the 12-month follow-up (CBT + SinHumo 37.1% vs. CBT + control App 42.6%). This pattern of results was similar at the end of treatment (CBT + SinHumo 68.6% vs. CBT + control App 62.8%) and at the 3-month (CBT + SinHumo 35.7% vs. CBT + control App 45.9%) and 6-month (CBT + SinHumo 35.0% vs. CBT + control App 41.2%) follow-ups, as no significant group differences were found.

Previous research analyzing the results of smartphone App-based smoking cessation interventions have shown mixed findings, as some studies have found higher smoking abstinence, whereas others did not find this effect.^11^ Results of studies combining apps and smoking cessation treatment are in the same line. For instance, Masaki et al.^17^ and Carrasco-Hernandez et al.^36^ found significantly higher abstinence rates in the App condition, whereas O´Connor et al.^18^ did not find statistically significant differences. These mixed results could be explained, at least in part, by the studies’ characteristics, making it difficult to establish comparisons between them. In the study of Masaki et al.,^17^ the app condition also included a web-based patient management software for physicians and a mobile CO-checker. Therefore, this intervention could be considered more complex because of the inclusion of other digital tools compared with the present and O´Connor et al.’s^18^ study. Regarding Carrasco-Hernandez et al.’s^36^ study, they used a pharmacotherapy-based intervention with CBT plus an App sending personalized notifications generated by artificial intelligence.

A plausible factor that could produce the nonsignificant group differences in our study is related to the fact that the comparison group received an intervention showing good abstinence rates at the 12-month follow-up in previous research.^24,37^ These results should be acknowledged, as high abstinence rates were reported in both groups at the 12-month follow-up. In this vein, previous systematic reviews have shown pooled abstinence rates at the 6-month follow-up or longer of a maximum of 25.6% for different smoking cessation interventions, including pharmacotherapy, counseling, behavioral interventions, or combined treatments.^3,4^

We also examined a minimum of 50% reduction in CPD from baseline to the 12-month follow-up, finding no significant group differences. This result is in line with previous research using mobile Apps for smoking cessation, showing an overall reduction of CPD from baseline to follow-ups but not differing between study conditions.^18,38–40^

Regarding engagement with the intervention, session attendance was similar between conditions and resulted from a significant predictor of abstinence outcomes at 12-month follow-up in both groups. This is in consonance with previous literature showing the importance of treatment attendance for improving smoking cessation outcomes.^41^

Regarding App use, the CBT + SinHumo App (experimental condition) had greater mean days of use than the control condition. Moreover, in the experimental condition, a higher number of days of SinHumo App use was a significant predictor of 12-month abstinence. These findings highlight the relevance of engagement with digital health technologies^42^ but also the key role of human support to improve Apps engagement.^43^

Lastly, the high levels of intervention satisfaction reported in both groups (*M* = 30.70 vs. *M* = 30.46, of a maximum of 32), and the absence of differences between conditions obtained are consistent with other studies such as that of O´Connor et al.^18^ in which an App was used combined with a smoking cessation intervention. In general, satisfaction with smoking cessation Apps is high even when they are fully automated.^38,44^

Overall, as Guo et al.^11^ highlight, even though using an App to quit smoking does not significantly improve abstinence rates, these digital tools could contribute to increasing access to effective smoking cessation interventions. In this vein, following a stepped-care approach,^45^ Apps could be included among the different interventions to quit smoking, providing different levels of intensity and delivery formats.

### Strengths and Limitations

Among the strengths of this study, we point out that the present randomized controlled trial is one of the few studies examining the long-term efficacy of combined treatments using Apps to quit smoking. We also included a large sample size of treatment-seeking people who smoke, and we obtained a high participant retention rate throughout the 1-year follow-up (91.0%). Including a control App (throughout the treatment and follow-up period) allowed us to control for the effect of using a digital tool during the intervention. Finally, our intervention was provided remotely, which could facilitate accessibility to treatment to people who smoke experiencing barriers such as geographical distance or work or family schedule incompatibilities.^46^

However, the limitations of this randomized controlled trial should also be considered when interpreting our findings. Firstly, biochemical verification for the abstinence outcome data was not conducted due to the remote nature of the intervention (through synchron video calls). This could have implications for the study results as a recent meta-analysis has shown that self-reported abstinence is higher compared to biochemically verified abstinence rates.^47^ Therefore, the percentages of abstinence could be misreported. However, other studies such as the one conducted by Webb et al.^48^ showed no statistically significant differences in quit rates between a group of a random sample of participants who underwent biochemical validation and those who only self-reported abstinence. To increase confidence in self-reported abstinence when conducting remote smoking cessation interventions, future research could include remote biochemical verification at least in a random sample of participants as in Webb et al.’s^48^ study. Secondly, in this study, we did not include a control condition with minimal intervention (eg, brief advice, counseling, and self-guided App) or a nonintervention group. For instance, studies comparing the efficacy of combined interventions (eg, CBT plus App) and fully automated Apps to quit are warranted. Thirdly, participants in this study were treatment-seeking people who smoke, so our findings cannot be generalized to those from the general population. Research on the specific characteristics of the people who smoke is needed to establish different intensity levels of smoking cessation interventions. For example, brief digital interventions could be adequate for non-dependent people who smoke, younger, or those not ready to quit,^49^ whereas tailored interventions may be necessary for people who smoke with serious psychiatric conditions.^50^ In this line, people who smoke with severe mental health disorders or other addictive disorders were excluded from the present study. Consequently, future research is needed to analyze the current intervention’s efficacy in these population groups, considering their specific characteristics.

## Conclusion

The inclusion of the SinHumo App through the manualized smoking cessation CBT-based intervention did not improve abstinence rates at the 12-month follow-up. However, abstinence rates and satisfaction with the intervention were high in both conditions. Remote smoking cessation interventions and complementary digital resources could increase the attractiveness and accessibility of traditional smoking cessation interventions.

## Supplementary Material

Supplementary material is available at *Nicotine and Tobacco Research* online.

ntae053_suppl_Supplementary_Table_1

## Data Availability

The data underlying this article will be shared on reasonable request to the corresponding author.

## Data Transparency

The data reported in this manuscript will be used to develop additional manuscripts. This manuscript analyzed the efficacy of an App combined with a cognitive-behavioral treatment to quit smoking through a randomized clinical trial. Additional planned manuscripts are currently being developed and not under review or published. We do not believe that these additional manuscripts constitute duplicate or piecemeal publications.
