# Supplementary material for: The Efficacy of the SinHumo App Combined With a Psychological Treatment to Quit Smoking: A Randomized Clinical Trial
Source: Nicotine Tob Res. 2024 Mar 27;27(3):429–37. doi: 10.1093/ntr/ntae053 (PMC11847783; doi:10.1093/ntr/ntae053)
Supplement: ntae053_suppl_Supplementary_Table_1 [file ntae053_suppl_supplementary_table_1.docx]

Supplemental Table 1. Self-reported Abstinence Rates by Treatment Condition using Multiple Imputation

|  | **Treatment Group** | | **Odds Ratio**  **(95% CI)** | ***p*** | **AOdds Ratio**  **(95% CI)** | ***p*** |
| --- | --- | --- | --- | --- | --- | --- |
|  | CBT+SinHumo App | CBT+ control App |  |  |  |  |
| Outcome variable | % (n) | % (n) |  |  |  |  |
| 7-day PPA at 12-month follow-up | 42.1 (59) | 45.3 (67) | 0.86 (0.53, 1.41) | .558 | 0.84 (0.51, 1.40) | .499 |
| 30-day PPA at 12-month follow-up | 37.1 (52) | 44.6 (66) | 0.74 (0.45, 1.22) | .237 | 0.71 (0.43, 1.19) | .194 |
| 7-day PPA at 6-month follow-up | 42.1 (59) | 45.9 (68) | 0.87 (0.53, 1.42) | .577 | 0.85 (0.51, 1.41) | .521 |
| 30-day PPA at 6-month follow-up | 42.1 (59) | 43.2 (64) | 0.96 (0.59, 1.58) | .875 | 0.95 (0.57, 1.57) | .832 |
| 7-day PPA at 3-month follow-up | 41.4 (58) | 49.3 (73) | 0.74 (0.45, 1.20) | .219 | 0.71 (0.43, 1.71) | .175 |
| End of treatment abstinence | 76.4 (108) | 65.5 (97) | 1.73 (1.02, 2.93) | .043 | 1.68 (0.97, 2.93) | .066 |

*Note:* CBT = Cognitive-Behavioral Treatment, ITT = Intention to Treat, PPA = Point Prevalence Abstinence, End of treatment abstinence = achieving at least 24 hours of abstinence in the last treatment session (session 8), OR = Odd Ratio, AOR = Adjusted Odd Ration, Smoking status coded as smoker = 0, abstinent = 1.
